# Region-specific remodeling of the enteric nervous system and enteroendocrine cells in the colon of spinal cord injury patients

**DOI:** 10.1038/s41598-023-44057-y

**Published:** 2023-10-06

**Authors:** Chloë Lefèvre, Camille Le Roy, Anne Bessard, Catherine Le Berre-Scoul, Justine Marchix, Emmanuel Coron, Marc Le Rhun, Charlène Brochard, Brigitte Perrouin-Verbe, Michel Neunlist

**Affiliations:** 1 Nantes Université, INSERM, CHU Nantes, IMAD, “The Enteric Nervous System in Gut and Brain Disorders”, 44000 Nantes, France; 2 Service de Médecine Physique et Réadaptation Neurologique, Nantes Université, CHU Nantes, 44000 Nantes, France; 3 Service de Gastroentérologie, Nantes Université, CHU Nantes, IMAD, 44000 Nantes, France; 4 CHU Rennes, Explorations Fonctionnelles Digestives, 35000 Rennes, France

**Keywords:** Diseases of the nervous system, Enteric neuropathies, Functional gastrointestinal disorders, Spinal cord diseases

## Abstract

Patients with spinal cord injury (SCI) suffer from major bowel dysfunction, whose exact pathophysiology, particularly the involvement of the enteric nervous system or epithelial dysfunction is poorly understood. Herein, we aimed to characterize the mucosal biopsies of the right and left colon in SCI patients vs controls (CT): (1) remodeling of key enteric neurotransmitters, (2) remodeling of enteroendocrine cells, and (3) mucosal inflammation compared to those in controls. In SCI, mucosal ACh concentration was lower in the right colon as compared to CT, but no change was observed in the left colon, and AChE expression was lower in both the right and left colons than in CT. While the VIP concentration was similar in the right and left colons, VIP mRNA expression was increased in the right colon and decreased in the left colon, in SCI patients as compared to CT. Interestingly, 5-HT concentration was reduced in the left colon but not in the right colon in SCI patients. Moreover, in SCI patients, as compared to CT, SERT mRNA expression was selectively increased in the left colon while TPH1 mRNA expression was increased in the right and left colons. Although mucosal TNFα and IL-1β mRNA expression did not significantly differ between SCI and CT groups, we identified a significant positive correlation between TNFα and IL-1β mRNA expression and left colon transit time in the SCI group. In conclusion, region-specific changes occur in the enteric neurotransmitter, serotonergic, and inflammatory pathways in the colon of SCI patients. The significant correlations between these pathways and clinical parameters in the left colon further set a scientific basis for designing therapeutic targets to improve colonic motor dysfunction in patients.

**Biobank information**: Spinal cord injury patients: PHRC ConstiCAPE—clinical trial NCT02566746. Controls: Anosain—clinical trial NCT03054415 and biobank of the “Institut des Maladies de l’Appareil Digestif (IMAD)” registered under number DC-2008-402.

## Introduction

Spinal cord injury (SCI) causes significant alterations to multiple organ systems. Beyond sensitive or motor impairments, impairments caused by SCI lead to autonomic dysfunction of the cardiovascular and respiratory systems, genito-sexual and bladder systems, and gastrointestinal tract^1^. The latter is also known as neurogenic bowel dysfunction (NBD). NBD is among the most prevalent disorders after SCI, affects up to 95% of patients^2^. Clinically, SCI patients most often suffer from delayed transit and defecation disorders, including anorectal dyssynergia and anal incontinence^3–6^. The management of NBD takes a significant amount of time on a daily basis and is ineffective due to a limited therapeutic arsenal^7^. In addition, NBD accounts for 15% of causes of rehospitalization after SCI^8^. Taken together, NBD has a major impact on the health and quality of life of SCI patients^9^. Indeed, improving bowel function is the second or third recovery priority for paraplegics and tetraplegics, even before the recovery of walking capacity^10^. Therefore, improving our understanding of the pathophysiological mechanisms underlying NBD is of major interest in the care of SCI patients.

Digestive dysfunction in SCI patients is related to the impairment of extrinsic sympathetic and parasympathetic innervation^11^. However, recent evidence suggests that the enteric nervous system (ENS) may be a major contributor to these dysfunctions. The ENS is an integrative and intrinsic nervous system embedded along the gastrointestinal tract that regulates digestive functions, such as motor functions (peristalsis, migrating motor complex), as well as intestinal epithelial barrier functions (secretion, permeability, cell proliferation, and differentiation)^12^. Alterations of the ENS occur in a wide range of digestive motility disorders, including slow transit constipation (STC)^13^, irritable bowel syndrome (IBS)^14^, as well as brain diseases such as Parkinson’s disease^15^ and autism spectrum disorders. In the latter, these alterations may contribute to digestive comorbidities observed in central nervous system disorders^16^. In SCI, changes in the ENS have only been scarcely characterized in patients. Only one study, to the best of our knowledge, showed the presence of myenteric neuronal cell loss and a reduction in calretinin-immunoreactive submucosal neurons in SCI patients^17^. However, animal models of SCI have highlighted the importance of ENS remodeling in SCI. In particular, similarly to human, a reduction in myenteric neurons density was reported in SCI-induced rats^18^. The changes in specific neurochemically-defined enteric neurons were described both in the ileum (decrease in the proportion of neuronal nitric oxide synthase neurons^19^) and in the colon (decrease in acetylcholine (ACh) concentration^20^ and nitrergic neurons^21^. Interestingly, in the colon, whose functions are largely affected in SCI patients, animal studies suggest region-specific changes in the ENS phenotype. In particular, Frias et al.^22^ reported a reduction in the smooth muscle contractility to muscarinic agonists in the distal but not in the proximal colon in rats. In contrast, in another study in rats, contractile response of colonic smooth muscle to bethanechol stimulation was affected in the proximal but not distal colon^20^. Thus, the changes in ENS phenotype could be associated with region-specific functional changes that are observed in SCI patients, with greater impairment in motility in the descending vs ascending colon^23–25^. These region-specific changes could reflect the fact that the right colon has a more important role in absorption of electrolyte and the left colon has an important role in storage and evacuation. However, whether region-specific changes in the ENS phenotype also occur in the colon of SCI patients and could contribute to these changes remains undetermined.

In addition to the ENS, enteroendocrine cells (EEC) are increasingly recognized as key cellular regulators of digestive functions. Indeed, EEC are a cellular subtype of the intestinal epithelial monolayer that act as mechanical and/or chemo sensors, triggering, upon stimulation, the release of mediators such as 5-hydroxytryptamine (5-HT) or cholecystokinin, which activate the ENS to modulate motility and/or epithelial secretory functions^26^. Among EEC, enterochromaffine cells (ECC), which synthesize 5-HT^27,28^, have gained particular attention in gut motility disorders. Indeed, EEC-derived 5-HT regulates gut motility^29–32^*.* Furthermore, specific deletion of ECC induces delayed gastric emptying and colonic motility^33^. Further, reduced expression of tryptophan hydroxylase 1 (TPH1), the rate-limiting enzyme in 5-HT synthesis, and serotonin reuptake transporter (SERT) has been reported in IBS patients with constipation^30,34^. Whether changes in ECC density and 5-HT synthesis pathways also occur in SCI patients remains unknown.

A major factor limiting pathophysiological understanding of both ENS and ECC dysfunctions in SCI patients relies on difficulties in accessing human tissues for both technical and ethical reasons. However, routine colonic mucosal biopsies have proven effective in studying both intestinal epithelial barrier function and the ENS phenotype in patients, particularly in Parkinson’s disease^35–37^. Therefore, this study combined similar approaches using mucosal biopsies of the right and left colons of SCI patients and controls to characterize (1) mucosal changes in ENS neurotransmitter expression, (2) changes in ECC density and 5-HT pathways, (3) mucosal inflammation, and (4) to identify putative correlations between biological and clinical parameters.

## Results

### Characteristics of SCI patients and CT

The samples from 15 CTs and 14 SCI patients were analyzed using immunoassay and transcriptomic studies. The patients’ characteristics are presented in Table 1. CTs were predominantly women (9/15; 60%), aged of 72.9 ± 14.9 years. SCI patients were predominantly men (11/14; 78.6%), younger with an age of 49.9 ± 14.7 years, p = 0.016. However, no statistical difference was observed among gender within each group. SCI patients had a majority of thoracic neurological levels (9/14; 64.3%) and a complete SCI (9/14; 57.1%). They had a delay post injury of 22.4 ± 15.6 years. Their mean colonic transit time was 92.8 ± 43.24 h (129.6 ± 10.18 in women and 85.44 ± 43.73 h in men). Their mean NBD score was 19.7 ± 7.1 points, corresponding to severe dysfunction. For the immunohistochemical study, mucosal samples from 13 CTs and 9 SCI patients were analyzed. The patient characteristics are described in Table 2.

**Table 1:** Characteristics of the patients used for PCR and immunoassay analyses.

		CT	SCl
		n = 15	n = 14
Gender (n (%))	Male	6 (40)	11 (78.6)
Age (mean ± SD)		72.9 ± 14.9	49.86 ± 14.73*
SCI (n (%))	Cervical level	–	5 (35.7)
	Thoracic level	–	9 (64.3)
Complete injury (%)		–	9 (64.3)
Delay post SCI (years, mean ± SD)		–	22.4 ± 15.6
Colonic transit time (hours, mean ± SD)		–	92.8 ± 43.24
NBD score (mean ± SD)		–	19.7 ± 7.1

*CT* control, *SCI* spinal cord injury, *n* number of patients, *SD* standard deviation, *NBD* neurogenic bowel dysfunction.

*Mann Whitney tests; p < 0.05.

**Table 2:** Characteristics of the patients used for immunohistochemical analysis.

		CT	SCl
		n = 13	n = 9
Gender (n (%))	Male	10 (76.9)	7 (77.8)
Age (mean ± SD)		65.6 ± 18.6	45.4 ± 16.1*
SCI (n (%))	Cervical level	–	3 (33.3)
	Thoracic level	–	6 (66.7)
Complete injury (%)		–	5 (55.5)
Delay post SCI (years, mean ± SD)		–	21.4 ± 13.4
Colonic transit time (hours, mean ± SD)		–	78.8 ± 40.6
NBD score (mean ± SD)		–	22 ± 4.6

*CT* control, *SCI* spinal cord injury, *n* number of patients, *SD* standard deviation, *NBD* neurogenic bowel dysfunction.

*Mann Whitney tests; p < 0.05.

### Analysis of mucosal neurotransmitters expression in SCI patients and CT

**Figure 1:**
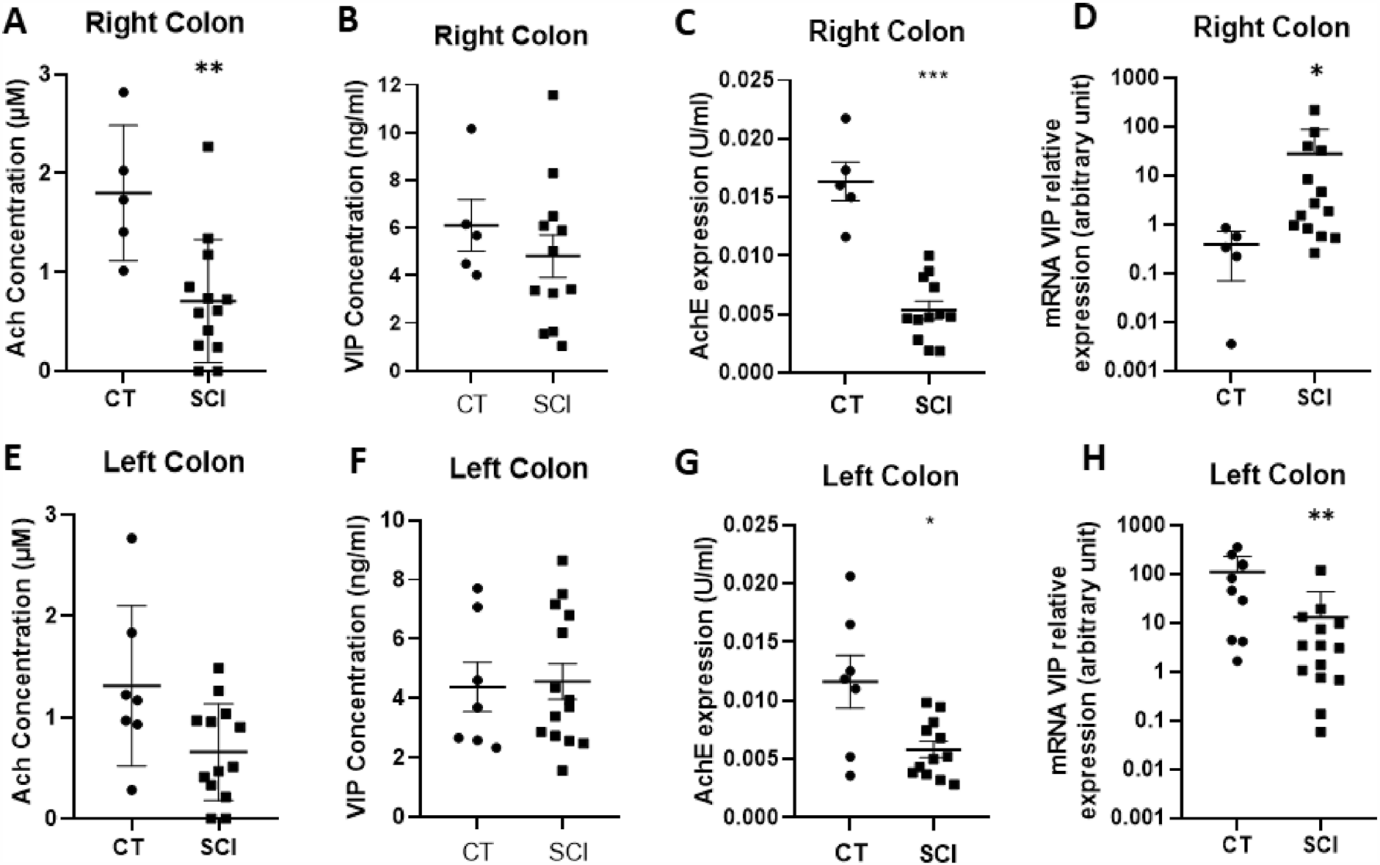
Impact of spinal cord injury (SCI) on neuromediators of the enteric nervous system. The acetylcholine concentration was significantly reduced in right colon of SCI patients (n = 13) (**A**) compared to that in controls (CT) (n = 5) (p = 0.007), but not in the left colon of SCI patients (n = 13) compared to those in CT (n = 7) (**E**). Acetylcholinesterase expression was decreased in both the right (n = 12) (**C**) and left (n = 12) (**G**) colon of SCI patients compared to those in CT (n = 5, right and n = 7, left) (respectively p = 0.004 and p = 0.028). The vasoactive intestinal peptide (VIP) concentration was unchanged in both the right (n = 12) (**B**) and left colon (n = 14) (**F**) of SCI patients compared to those in CT (n = 5, right and n = 7, left). However, mRNA VIP expression was significantly higher in the right colon (n = 14) (p = 0.0103) (**D**) but significantly lower in the left colon of SCI patients (n = 14) (p = 0.003) (**H**) compared to those in CT (n = 5, right and n = 9 left).

We first determined whether two key enteric neurotransmitters involved in ENS regulation of gastrointestinal functions, ACh and VIP, were altered in mucosal biopsies of SCI patients compared to that of CTs. The ACh concentration was significantly reduced by 39% in the right (p = 0.007) but not in the left colon of SCI patients, as compared to those of CTs (Fig. 1A and E). Interestingly, AChE expression was significantly reduced by 67% in the right colon (p = 0.004) and by 54% in the left colon (p = 0.028) as compared to that of CTs (Fig. 1C and G). The VIP concentration did not change in the mucosal biopsies of SCI patients as compared to that in CTs in both the right and left colons (Fig. 1B and F). However, we observed a region-specific differential regulation of VIP mRNA expression (Fig. 1D and H). VIP mRNA expression was significantly increased in the right colon (p = 0.010) and significantly decreased in the left colon (p = 0.003) of SCI patients compared to those in CTs.

### Analysis of colonic serotoninergic mucosal pathways in SCI patients and CT

Then, we aimed to determine whether serotoninergic pathways, previously reported to be altered in colonic motility disorders, were also affected in SCI patients as compared to CTs.

#### Enteroendocrine cells counts in mucosa of SCI patients and CTs

**Figure 2:**
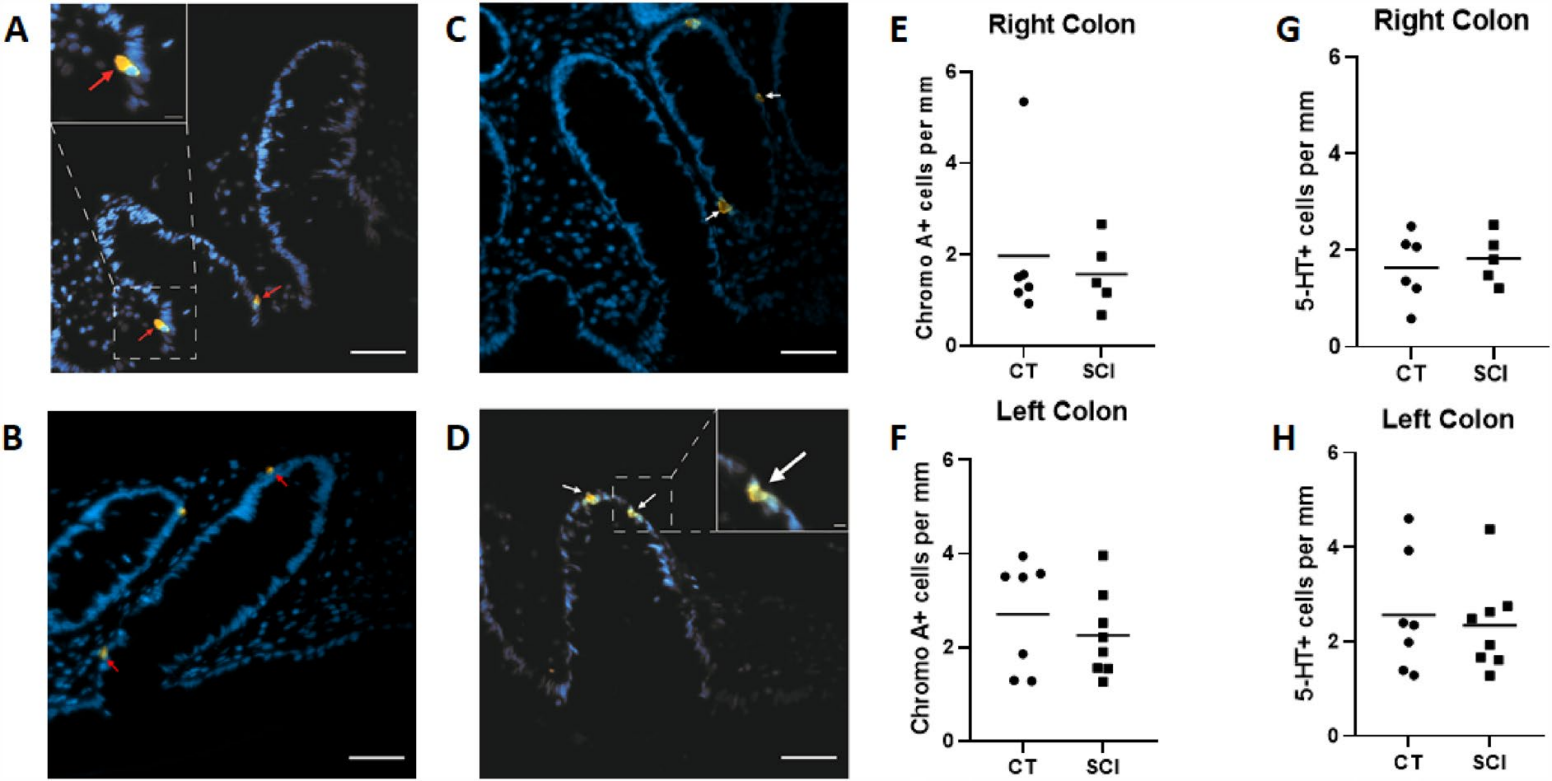
Impact of spinal cord injury (SCI) on enteroendocrine cells (EEC). Immunochemical staining of paraffin-embedded section of colonic mucosa against Chromogranin A ((**A**,**B**) red arrows) and against 5-hydroxytriptamine 5-HT ((**C**,**D**) white arrows) associated with a marker of the cell nuclei (DAPI, in blue); scale bar = 50 µm. Higher-magnification micrographs (white boxed regions; scale bar = 10 µM (**A**) or 5 µM (**D**)). There was no difference between EEC density regarding the crypt perimeter (cellular linear density) in SCI patients in both the right (n = 5; (**E**)) and left colons (n = 8; (**F**)) and CT (n = 6, right; n = 7, left). There was no difference in 5-HT + cells density per mucosal length compared with CT (right, n = 6; left, n = 7) in both the right (n = 5; (**G**)) and left colons (n = 8; (**H**)) of SCI patients.

First, we analyzed the morphological characteristics of the colonic mucosa of SCI patients and CTs. The mean height of colonic crypt was similar in SCI patients and CTs for both the right (136.8 ± 39.4 vs. 181.5 ± 107.3 µm, respectively; p = 0.662) and left colons (160.9 ± 52.0 vs. 173.0 ± 49.0 µm, respectively; p = 0.792). Next, we characterized the mucosal density of ECC using an antibody against ChgA. All microphotographs were taken with the same exposure time. The number of ChgA-IR cells per mucosal length (Fig. 2A,B,E,F) did not significantly differ between SCI patients and CTs, in both the right (2.0 ± 1.7 vs. 1.6 ± 0.8 cells per mm, p = 0.931, respectively) and left colons (2.7 ± 1.2 vs. 2.3 ± 0.9 cells per mm, p = 0.152, respectively).Then, we analyzed the mucosal ECC density in SCI patients and CTs using an anti-5-HT antibody (Fig. 2C,D,G,H). We identified no difference in the number of 5-HT-IR cells per mucosal length between SCI patients and CTs in the right (1.6 ± 0.7 vs .1.8 ± 0.5 cells per mm p = 0.662, respectively) and left colons (2.6 ± 1.3 vs. 2.3 ± 1.0 cells per mm p = 0.955, respectively).

#### Mucosal 5-HT concentration and mRNA expression of TPH-1 and SERT in SCI patients and CTs

**Figure 3:**
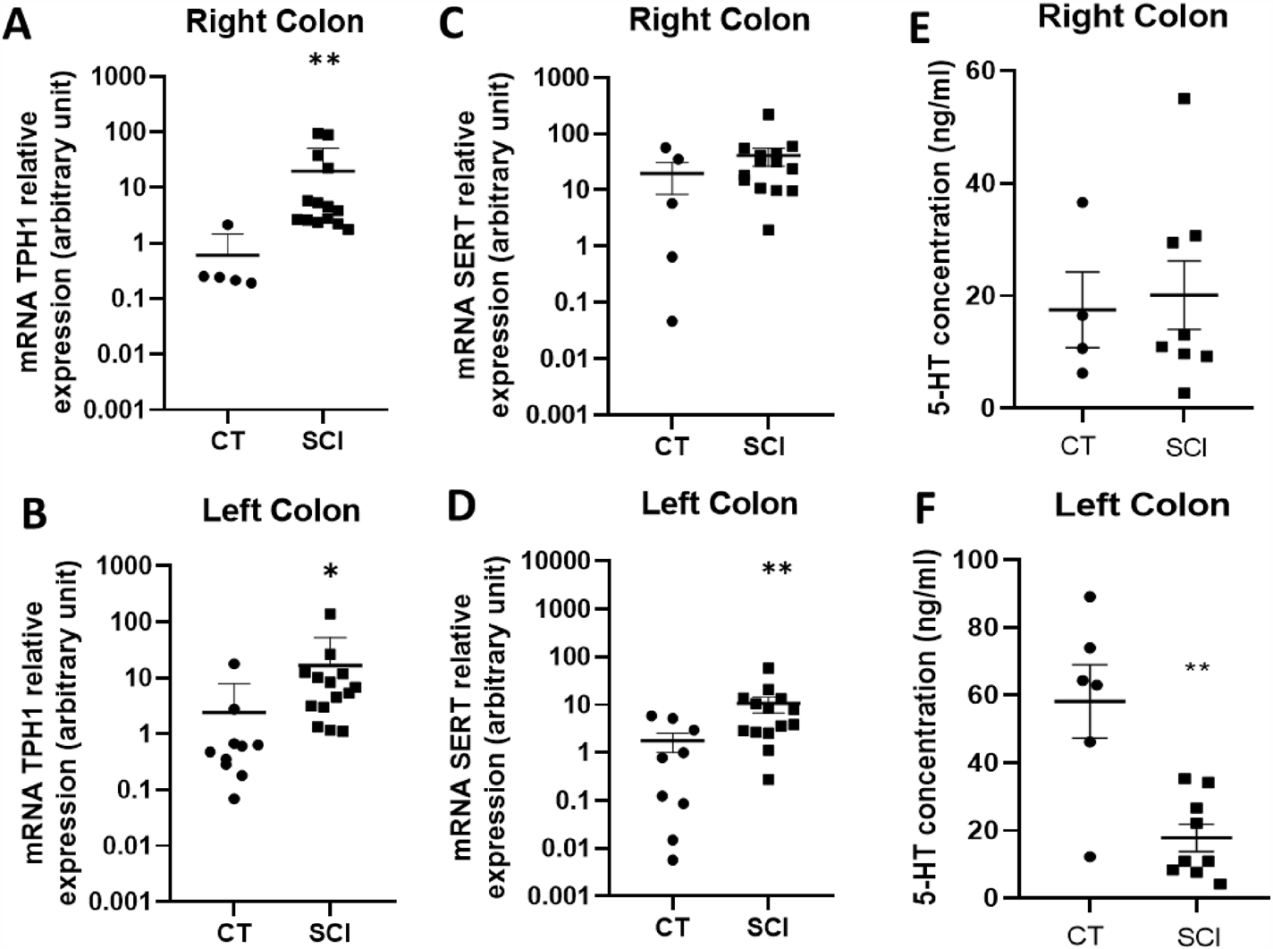
Impact of Spinal Cord Injury (SCI) on mucosal serotoninergic pathways. mRNA Tryptophan hydroxylase 1 (TPH1) expression was higher in both the right (n = 14) (**A**) and left (n = 14) (**B**) colons of SCI patients compared to that in controls (CT) (n = 5, right; n = 10, left) (respectively p = 0.002 and p = 0.019). The mRNA Serotonin Reuptake Transporter (SERT) expression increased in the left colon (n = 14) of SCI patients compared to that in CT (n = 9, left; n = 5, right) ((**D**), p = 0.007) but not in the right colon (n = 14) (p = 0.257) (**C**). Moreover, 5-hytroxytriptamine (5-HT) concentration was unchanged in the right colon (n = 8) **(E)** but lower in the left colon (n = 9) (**F**) of SCI patients compared to that in CT (n = 4, right; n = 6, left) (p = 0.005).

Subsequently, we characterized the 5-HT concentration and expression of key molecules involved in serotonin synthesis and reuptake, i.e. TPH1 and SERT, respectively (Fig. 3).

First, we showed that the mucosal 5-HT concentration in the right colon was similar in patients as compared to CT (Fig. 3E, p = 0.999). However, in the left colon, there was a significant decrease in the mucosal concentration of 5-HT in SCI patients as compared to CT (Fig. 3F, p = 0.005). TPH1 mRNA expression was significantly increased in both the right colon (p = 0.002) (Fig. 3A) and the left colon (p = 0.019) (Fig. 3B) as compared to CT. Interestingly, SERT mRNA expression was not modified in the right colon (Fig. 3C, p = 0.257) but it was significantly increased in the left colon (Fig. 3D, p = 0.007), as compared to CT.

### Mucosal colonic inflammation in SCI patients and CT

We further aimed to determine whether changes in ENS and ECC were associated with changes in the mucosal expression of key inflammatory mediators, such as IL-1β and TNFα.

**Figure 4:**
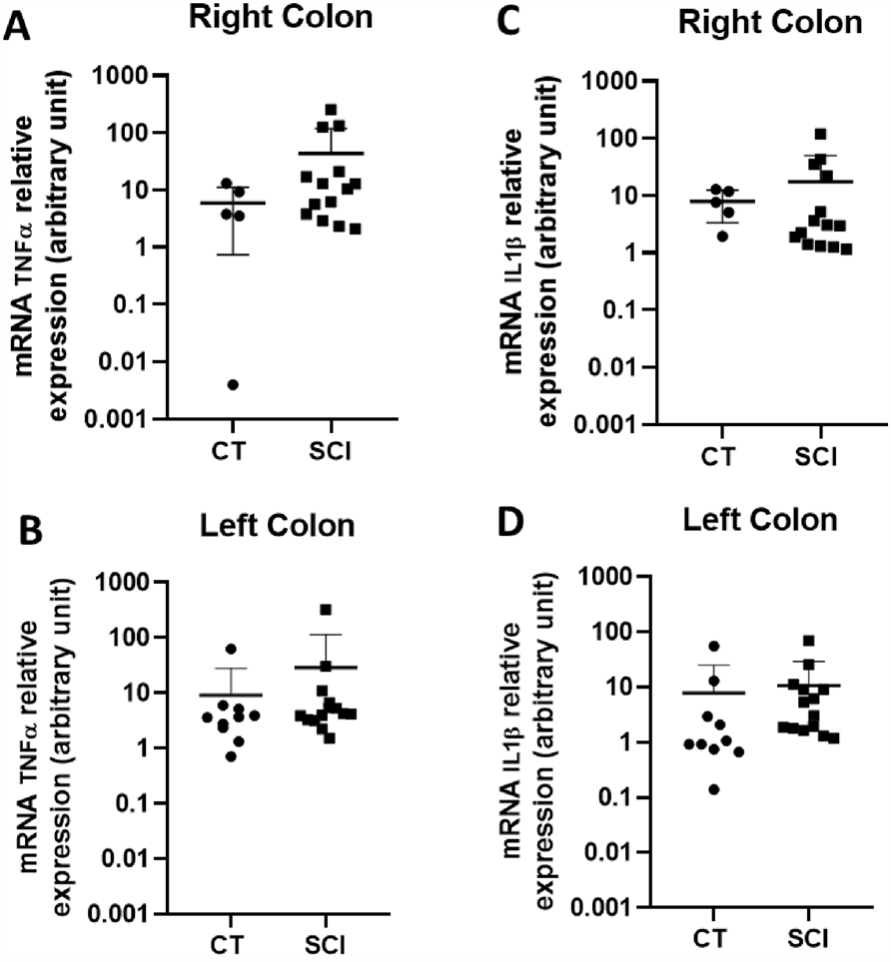
Impact of Spinal Cord Injury (SCI) on local inflammation. No significant increase occurred in TNFα mRNA expression in SCI patients (n = 14) compared with CT both in the right colon (CT n = 5) (**A**) and in the left colon (CT n = 10) (**B**). IL-1ß mRNA expression tended to increase in the left colon in SCI patients compared to those in CT (p = 0.056) (**D**), but not in the right colon (**C**).

IL-1β mRNA expression tended to be increased in the left colon in SCI patients as compared to CT (p = 0.056) (Fig. 4C), but not in the right colon (p = 0.444) (Fig. 4D). There was no significant increase in TNF-α mRNA expression compared to CT in the right colon (p = 0.257) (Fig. 4A) nor in the left colon (p = 0.213) (Fig. 4B). Next, we aimed to identify the putative correlation between the ENS, markers, and inflammatory mediators in the SCI group. We found that IL-1β expression was positively correlated with TPH1 mRNA expression in both the right and left colon (r = 0.567 and r = 0.776, respectively; p = 0.029 and p = 0.002, respectively) (Supplementary Fig. 1A and B, respectively). TNFα mRNA expression was also positively correlated with TPH1 mRNA expression in both the right and left colon (r = 0.671 and r = 0.587, respectively; p = 0.010 and p = 0.024, respectively) (Supplementary Fig. 1C and D, respectively). No correlation was observed between IL-1β or TNF-α and 5-HT or SERT nor between IL-1β or TNFα expression and VIP or ACh concentration.

### Correlations between clinical and biological parameters in SCI patients

Finally, we sought to identify putative correlations between the biological parameters in the right and left colon (enteric neurotransmitter expression, serotoninergic pathways, mucosal cytokine expression) and the clinical parameters of SCI patients: total colonic transit time, right colonic transit time, left colonic transit time (i.e., left and sigmoid colonic transit time), NBD score, and Kess score. The correlations are summarized in Tables 3 and 4.

**Table 3:** Correlations between total (A) and segmental (B-C) transit time and biological parameters.

Correlations		Neurotransmitters			Serotoninergic pathways			Mucosal inflammation		
			r	p-value		r	p-value		r	p-value
A. Total transit time	1 Right colon	Ach	0.142	0.328	5-HT	0.072	0.436	TNFα	− 0.109	0.374
		AChE	0.256	0.223	TPH1	− 0.4	0.099	IL1-β	− 0.027	0.470
		VIP	− 0.246	0.231	SERT	− 0.301	0.183			
	2 Left colon	Ach	0.415	0.090	5-HT	0.578	0.070	TNFα	0.601	0.027*
		AChE	0.406	0.107	TPH1	0.279	0.202	IL1-β	0.774	0.003**
		VIP	0.116	0.360	SERT	− 0.584	0.031*			
B. Right colon transit time	Right colon	Ach	0.111	0.364	5-HT	0.036	0.469	TNFα	− 0.023	0.476
		AChE	0.301	0.183	TPH1	− 0.235	0.229	IL1-β	0.16	0.319
		VIP	− 0.237	0.240	SERT	− 0.142	0.338			
C. Left colon transit time	Left colon	Ach	0.418	0.088	5-HT	0.643	0.048*	TNFα	0.601	0.027*
		AChE	0.46	0.078	TPH1	0.296	0.187	IL1-β	0.656	0.016*
		VIP	0.227	0.191	SERT	− 0.492	0.063			

Enteric neurotransmitters [Acetylcholine (ACh) concentration, acetylcholinesterase expression (AChE), Vaso intestinal peptide (VIP) mRNA expression], serotoninergic pathways [Serotonin (5-HT) concentration, Tryptophan hydroxylase 1 (TPH1) mRNA expression, Serotonin transporter (SERT) mRNA expression], and mucosal inflammation [Tumor Necrosis Factor alpha (TNFα) and Interleukin -1 beta (IL-1β) mRNA expression], in the right (**A1** and **B**) and left colons (**A2** and **C**) of spinal cord injury (SCI) patients. Spearman’s one-tailed correlations; *p < 0.05; **p < 0.01, n = 13 SCI patients.

**Table 4:** Correlations between NBD score (A) or Kess score (B) and biological parameters.

Correlations		Neurotransmitters			Serotoninergic pathways			Mucosal inflammation		
			r	p-value		r	p-value		r	p-value
A. NBD score	Right colon	ACh	− 0.102	0.375	5-HT	− 0.204	0.316	TNFa	0.411	0.092
		AChE	− 0.183	0.284	TPH1	0.586	0.019*	IL1-B	0.337	0.141
		VIP	0.397	0.100	SERT	0.313	0.160			
	Left colon	Ach	− 0.394	0.091	5-HT	0.108	0.404	TNFa	− 0.243	0.222
		AChE	− 0.394	0.102	TPHI1	− 0.056	0.432	IL1-6	− 0.211	0.254
		VIP	− 0.39	0.105	SERT	0.155	0.315			
B. Kess score	Right colon	Ach	0.114	0.360	5-HT	− 0.301	0.233	TNFa	0.224	0.240
		AChE	0.105	0.372	TPHI1	0.499	0.042*	IL1-6	0.077	0.406
		VIP	0.193	0.272	SERT	− 0.463	0.065			
	Left colon	Ach	− 0.454	0.060	5-HT	− 0.036	0.472	TNFa	− 0.414	0.090
		AChE	− 0.316	0.157	TPH1	− 0.589	0.024*	IL1-8	− 0.28	0.187
		VIP	− 0.396	0.101	SERT	− 0.025	0.471			

Enteric neurotransmitters [Acetylcholine (ACh) concentration, acetylcholinesterase expression (AChE), Vaso intestinal peptide (VIP) mRNA expression], serotoninergic pathways [Serotonin (5-HT) concentration, Tryptophan hydroxylase 1 (TPH1) mRNA expression, Serotonin transporter (SERT) mRNA expression], and mucosal inflammation [Tumor Necrosis Factor alpha (TNFα) and Interleukin -1 beta (IL-1β) mRNA expression], in the right and left colons of spinal cord injury (SCI) patients. Spearman’s one-tailed correlations; *p < 0.05, n = 13 SCI patients.

#### Enteric neurotransmitters

**Figure 5:**
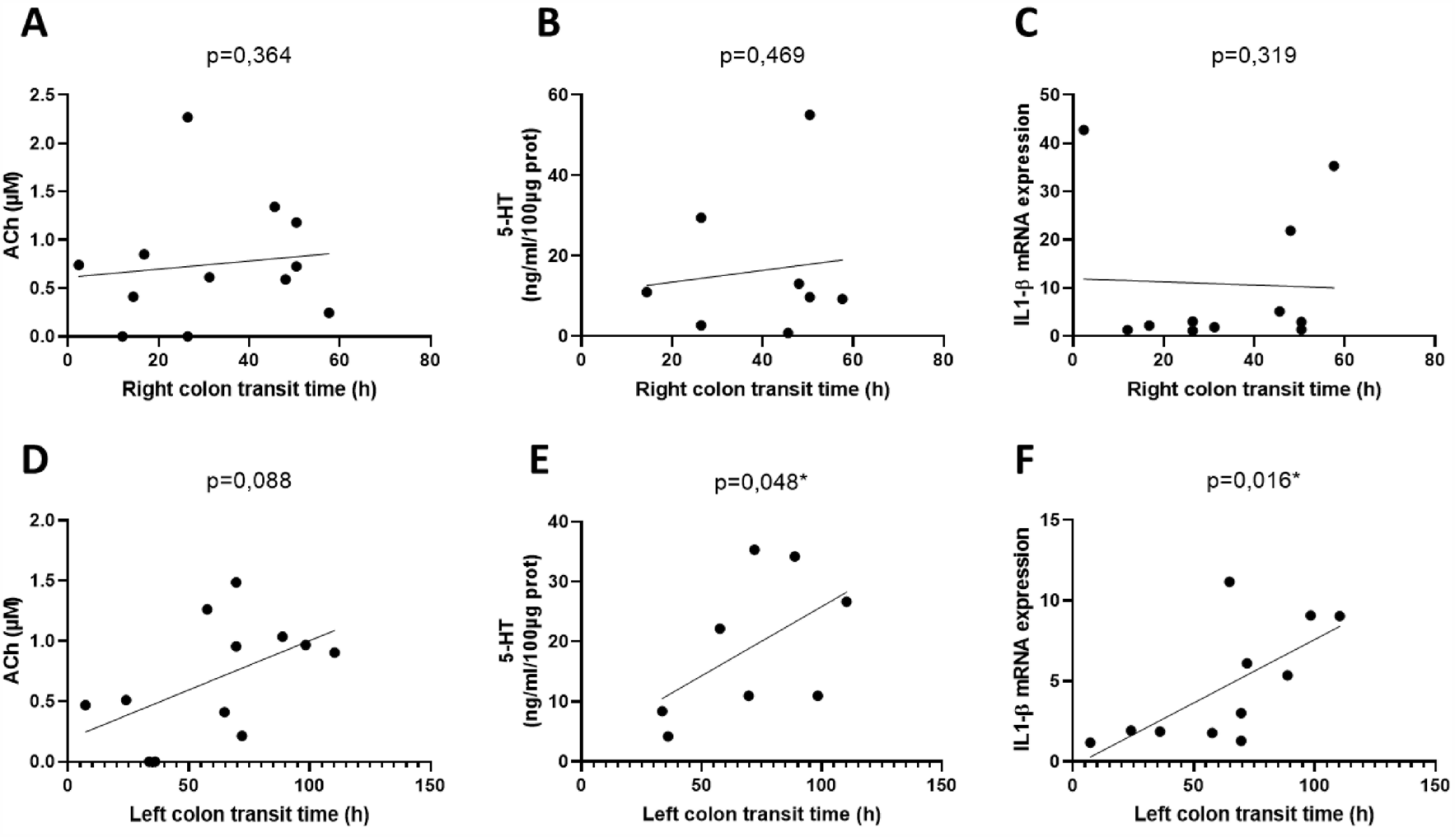
Clinico-biological correlations between transit time and biological parameters in the right (**A–C**) and left colon (**D**–**F**) of spinal cord injury patients. The represented biological parameters are: Acetylcholine (ACh) concentration in right colon (**A**) and left colon (**D**); Serotonin (5-HT) concentration in right colon (**B**) and left colon (**E**); Interleukin-1 beta (IL-1β) mRNA expression in the right colon (**C**) and left colon (**F**). Spearman’s one-tailed correlation coefficient (n = 13 spinal cord injury patients).

We first correlated the concentration and mRNA expression of mucosal enteric neurotransmitters (ACh, VIP, and AChE expression) with clinical parameters in SCI patients. No significant correlation was found between these parameters in either organ (Tables 3 and 4; Fig. 5A and D).

#### Serotoninergic pathways

Next, we correlated 5-HT concentration and TPH1 and SERT mRNA expression with clinical parameters. Interestingly, we found a significant correlation between mucosal 5-HT concentration and left colon transit time (r = 0.643, p = 0.048, Fig. 5B) as well as a trend for a correlation with the total transit time (r = 0.578; p = 0.07, Table 3), but not in the right colon (Fig. 5E, Table 3). Additionally, in the left colon, SERT mRNA expression negatively correlated with the total transit time (r = − 0.584; p = 0.031) (Table 3).

While TPH1 mRNA expression and transit time exhibited no correlation, TPH-1 mRNA expression positively correlated with the NBD score in the right colon (r = 0.586; p = 0.019) but not in the left colon (r = − 0.056; p = 0.432). Interestingly, TPH1 mRNA expression positively correlated with the Kess score in the right colon (r = 0.499; p = 0 > 0.42) and negatively in the left colon (r = − 0.589; p = 0.024).

No correlation was observed between 5-HT concentration, TPH1, or SERT mRNA expression in the right or left colon, and the other clinical parameters.

#### Mucosal inflammation

Finally, we correlated mucosal cytokine mRNA expression in the right and left colons using clinical parameters. Interestingly, in the left but not in the right colon, IL-1β mRNA expression significantly correlated with both the total (r = 0.774; p = 0.003) and left colon transit times (r = 0.656; p = 0.016) (Fig. 5C and F). Similarly, in the left but not in the right colon, TNFα mRNA expression significantly correlated with the total (r = 0.601, p = 0.027) and left colon transit times (r = 0.601, p = 0.027).

Also, we did not find any correlation between TNFα and IL-1β mRNA expression in the right or left colon and the other clinical parameters, such as the NBD or Kess score (Table 4).

## Discussion

In this study, we aimed to (1) identify the remodeling of mucosal enteric neurotransmitters and serotoninergic pathways in living SCI patients as compared to those in the CTs and (2) to correlate these changes with the bowel motor dysfunctions observed in these patients. First, we demonstrated the existence of region-specific changes in enteric neurotransmitters and serotoninergic pathways in SCI patients. Second, serotoninergic pathways as well as inflammatory cytokines in the left colon, but not in the right colon, were significantly correlated with transit time in the left colon, suggesting that targeting these pathways in colon-specific regions could improve motility dysfunction in SCI patients.

The first major finding of this study was to provide a comprehensive analysis of key neuromediators (ACh, VIP, and 5-HT) that could contribute to gut motility dysfunction in SCI patients. We found region-specific changes in neuromediator concentrations in the right and left colons of SCI patients as compared to those of CTs. In particular, we identified a significant and region-specific decrease in the mucosal concentration of ACh in the right colon and lower 5-HT concentration in the left colon as compared to that of CTs. A similar reduction of 5-HT was reported in a model of rat SCI^38^. In addition, we also observed an increased SERT mRNA expression, which could reduce the bioavailability of 5-HT and further contribute to constipation in SCI patients. The modified mucosal 5-HT levels have been associated with colonic constipation in diseases such as IBS^34^ and Slow transit constipation^30^. Conversely, the increased expression of TPH1 may be considered as an adaptive response of the gut to low levels of 5-HT. Interestingly, these changes in 5-HT were not associated with changes in ECC density, but rather with the modulation of 5-HT expression. One of the potential factors involved in the regulation of TPH1 expression in both colons could be IL-1β as we found a positive linear correlation between IL-1β and TPH1 mRNA expression. However, the ability of IL-1β to regulate TPH1 mRNA expression remains unknown. The importance of this altered 5-HT in gastrointestinal motility disturbances is also further highlighted by the fact that therapeutic approaches for improving motility in preclinical models of SCI, such as electroacupuncture^38^ or microbiota transfer^39^, are associated with the restoration of 5-HT levels. Moreover, 5-HT4R agonists such as prucalopride^40^ demonstrably improve colonic transit time in SCI patients. Surprisingly, we found an inverse correlation between 5-HT level and transit time in the colon. This counterintuitive observation may indicate that the severity of constipation could induce a response in the gut to increase 5-HT production to counteract the motility deficit.

In addition to changes in 5-HT, we observed a decrease in mucosal ACh concentration in SCI patients, but only in the right colon. Interestingly, this decrease in ACh concentration was previously reported in the literature in a rat model of chronic SCI^21^. In this model, the decrease of ACh was also region-specific in the right colon but not in the left^20^. Interestingly, this decrease in ACh was associated with a reduction in AChE expression, which could dampen the loss of cholinergic components by enhancing ACh bioavailability. Alternatively, this reduction in ACh and AChE could reflect a loss of mucosal innervation by cholinergic neurons that also synthetize AChE. A loss of myenteric neurons associated with a decrease in nerve fiber density has been reported in the myenteric plexus of both patients with chronic SCI^17^ and animal models of SCI^18^. The functional consequences of these changes on mucosal homeostasis, such as permeability or antimicrobial defense, remain unexplored. Interestingly, a recent report on Hirschsprung’s disease revealed that reduced cholinergic mucosal innervation was associated with an increased risk of enterocolitis^41^. This may be a result of the reduced ability of cholinergic pathways to secrete mucus or antimicrobial peptides, which both act as a defense mechanism of the intestinal epithelial barrier. Interestingly, animal models of SCI have shown an increased intestinal paracellular permeability in mice 7 days post SCI^42^. Supporting the hypothesis of an altered intestinal barrier integrity in humans, is the observation that serum I-FABP and zonulin levels are increased in chronic SCI patients as compared to controls^43^. Finally, although the ACh content was reduced, no change was observed in the mucosal content of another key neuromediator involved in gut physiology, such as VIP. However, the VIP mRNA expression was increased in the right colon and decreased in the left colon. These results remain unexplained and may reflect the fact that, besides enteric neurons, other cells of the mucosal microenvironment, such as immune cells, can produce VIP^44^ and are differentially regulated.

Another important finding of our study was the significant correlations between clinical parameters (colonic transit time; NBD score) and biological parameters, mainly in the left colon, suggesting that these interactions could drive motility disorders in SCI patients and may be considered as putative therapeutic targets. We found a linear positive correlation between both TNFα and IL-1β mRNA expression in the left colon and total transit time. This suggests that gut inflammation can contribute to motility disorders or function as a marker of motility disorders in SCI patients. Although we did not measure a significant increase in IL-1β or TNFα, only a trend for IL-1β, in the left colon in SCI patients as compared to CT patients, this absence could result from the limited number of patients and/or cellular variability of single biopsies. However, inflammation has been previously shown to contribute to motility dysfunctions reminiscent of those observed in SCI in patients with STC or Parkinson Disease^45^. Therefore, targeting intestinal inflammation may help improve motility dysfunction in SCI patients.

This study also had some limitations. First, our findings require further confirmation in a validation cohort because of the relatively small number of patients. The results, especially concerning correlation analyses, are considered exploratory to guide future research. In particular, a longitudinal cohort could allow us to identify the time window of appearance of these lesions and whether targeting these changes earlier could prevent or reduce the development of gut motility disorders. Second, we used two independent cohorts to increase the number of controls included in our study, including biopsies of healthy controls and mucosal samples obtained at distance from colorectal tumor. Concerning the latter cohort, although tissues were taken at distance from the tumor, one cannot fully rule out the influence on the tumor upon parameters studied. In addition, a potential confounding factor of our study is that SCI patients were significantly younger than controls. Age-dependent decrease of neuromediator expression such as ChAT^46^ and increase of EEC density have been reported in aging patients^47^. However, our results show inverse results to what would have been expected if age would have been a cofounding factor.

Furthermore, the use of functional approaches relying on high-resolution manometry or exploration of gut permeability may improve our ability to identify the contribution of specific mediators to the functional changes observed in SCI patients. One should also take into considerations that changes in mucosal enteric mediators might not mirror the changes occurring in the myenteric plexus which is responsible for the regulation of motor functions. Although reduced expressions of ACh and ChAT were reported in the human colon during constipation^48,49^, no correlation was found between the mucosal ACh concentration, AChE expression, and colonic transit time. In addition, besides changes in Ach and VIP, dysfunctions of other mediators (such as nitrergic, purinergic or tachykininergic) or their receptors, as described for Ach^22^ could contribute to SCI associated motor dysfunctions. Altogether these limitations warrant that further studies characterizing changes in ENS and enteroendocrine remodeling in SCI patients are required in the future.

In conclusion, our findings illustrate major changes in the expression of key enteric neuromediators involved in the regulation of mucosal functions as well as major modifications of the enteric endocrine system. The functional consequences of the gut pathophysiological processes observed in SCI patients remain to be determined, as well as whether targeting these changes may improve bowel dysfunction in SCI patients.

## Methods

### Patients

Fourteen SCI patients who were included in a clinical trial (PHRC ConstiCAPE clinical trial NCT02566746) from July 2016 to October 2020 were enrolled in the study. This study aimed to evaluate the effect of percutaneous endoscopic cecostomy (PEC) on the quality of life of SCI patients. Colonic functional exploration was performed by evaluating the initial colonic transit time using 10 radiopaque markers ingested every morning for 6 consecutive days, then on the 7th day an abdominal X-ray was performed to calculate total and segmental (right, left, and rectosigmoid) colonic transit time according to Danquechin method^50,51^. Patients completed several forms, including the NBD score, which is a validated score for evaluating digestive function in SCI^52^. Patients underwent pre-inclusion colonoscopy with standard mucosal colonic biopsies before treatment modification and PEC.

The exclusion criteria were: inability to provide informed consent (guardianship, curatorship), known hemostasis disorders, severe obesity, history of colic resection surgery, or inflammatory bowel disease.

Two control (CT) groups were included: the first CT group consisted of healthy volunteers from a clinical trial (Anosain—clinical trial NCT03054415)^53^, from May to September 2017, used for immunoassay. The second, used for immunohistochemistry, transcriptomic and protein analysis, was obtained from a historical cohort of patients who underwent colorectal tumor resection, wherein segments of the colon were collected at a distance from the tumor in a healthy area. This historical cohort of the “Institut des Maladies de l’Appareil Digestif (IMAD)” is registered under the number DC-2008–402. Written informed consent for participation in the trial or biobank was obtained from each participant prior to inclusion.

### Tissue conditioning

Biopsies were performed on the right and left colon of SCI patients. For the CT “Anosain,” biopsies were collected on left colon and for the biobank IMAD, we used tissue biopsies taken from fixed right or left colon. Biopsies or tissue fragments were placed immediately in sterile Hank’s balanced salt solution maintained at 4 °C on ice and immediately transported to the laboratory. Five biopsies were obtained: one biopsy was placed in radioimmunoprecipitation assay (RIPA) buffer for protein analysis, two in RNA lysis buffer (RA1) for transcriptomic analysis, and two for immunochemistry analysis.

### ACh assay

Biopsies were lysed in RIPA lysis buffer (Millipore, Burlington, MA, USA) containing sodium orthovanadate (Sigma-Aldrich, USA), phosphatase inhibitors (Phosphatase inhibitor cocktail 3; Sigma-Aldrich, USA), and protease inhibitors (Complete; Roche Diagnostics, France) using a Precellys 24 tissue homogenizer (Bertin Technologies, France), followed by sonication with a Vibracell 75,186 (Sonics, USA). Total protein levels were quantified using an Eppendorf Biophotometer (France) with Bradford solution (Sigma-Aldrich, USA). ACh concentration and acetylcholinesterase expression (AChE) were determined in tissue homogenates containing equal amounts of protein (100 µg) (Amplex red ACh/AChE assay kit; Thermo Fisher).

### 5-HT assays

Serotonin concentration in biopsies was assessed using a serotonin ELISA kit (E4294; Biovision, Milpitas, USA) according to the manufacturer’s protocols. Biopsies lysed in RIPA buffer were used to assess 5-HT release. The plate was washed two times with 1 × wash solution, and then samples were added and incubated with biotin-detection antibody working solution for 45 min at 37 °C. Following incubation, the liquids were discarded, the wells were manually washed three times, each with the diluted wash solution, and horseradish peroxidase was added to each well. Following incubation at 37 °C for 30 min, the wells were washed again, and tetramethylbenzidine substrate was added to all wells and incubated for 15 min at 37 °C in the dark. The reaction was terminated by adding a stop solution, and the color changed from blue to yellow. The color change was measured at 450 nm using a spectrofluorometer (Biotek, France). The data were calculated for 100 µg protein.

### Quantitative polymerase chain reaction (PCR) analysis

Total RNA was extracted from biopsies placed in RA1 buffer with a Precellys 24 tissue homogenizer (Bertin Technologies) using Nucleospin RNA II (Machery-Nagel, France) according to the manufacturer’s instructions. Potential genomic DNA contamination was removed by treatment with Turbo DNase (Ambion Inc., USA), and RNA was quantified using a Nanodrop 200° ND-1000 UV–vis spectrophotometer (Nanodrop Technologies, USA). The reverse transcriptase reaction was performed using 1 µg total RNA incubated at 72 °C for 3 min using the Super Script III Reverse Transcriptase System kit (Thermo Fisher Scientific, France) in a Thermal Cycler 2720 thermocycler (Applied Biosystems, USA) (25 °C for 5 min, 50 °C for 55 min, 70 °C for 15 min). PCR amplification was performed using an Absolute Blue SYBR Green Fluorescein Kit (Roche Molecular Biochemicals, France) and run on a StepOnePlus system (Life Technologies). The studied genes were vasoactive intestinal peptide (VIP), TPH1, tumor necrosis factor alpha (TNFα), and interleukin 1 beta (IL-1β).

- VIP # NM_003381, forward: 5’-CGGCATGGCCTCTTTACAGGGC-3’; reverse: 5’-ACTCCATCAGCATGCCTGGCA-3’
- TPH1 # NM_004179, forward: 5’-ATACCCCAGAGCCAGATACC-3’; reverse: 5’- GTAGCACGTTGCCAGTTTTTG-3’
- TNFα # NM_000594, forward: 5’- CCCGAGTGACAAGCCTGTAG-3’; reverse: 5’- TGAGGTACAGGCCCTCTGAT-3’
- IL-1β # NM_000576.2, forward: 5’-GAGCAACAAGTGGTGTTCTCC-3’; reverse: 5’- TTGGGATCTACACTCTCCAGC-3’

The expression of S6 ribosomal protein was used as a reference gene: RPS6 # NM_001010.2, forward:5’-AAGCACCCAAGATTCAGCGT; reverse:5’- TAGCCTCCTTCATTCTCTTGGC-3’.

For SERT gene expression, PCR amplification was performed using TaqMan primers Hs00169010_m1 and RPS6 control (Hs04195024_g1) (Thermo Fisher Scientific, France).

The relative expression of gene interest was measured by the 2^-ΔΔCt^ method. Samples with a cDNA expression level outside the standard curve were excluded from the analysis.

### Immunohistochemical analysis

Biopsies were pinned and stretched in a dissection dish, then fixed in 0.1 M phosphate buffered saline (PBS) containing 4% paraformaldehyde for 3 h at room temperature. After three PBS washes, the mucosa was first collected under a microscope and stored in PBS/NaN_3_ (0.1%). For 5 SCI patients, tissue conservation was poor, making them unusable for immunohistochemistry.

The mucosa was embedded in paraffin and microtome-sectioned (3-µm thick) mounted on glass slides. Sections were deparaffinized by incubating the slides in two successive baths of xylene (2 × 7 min), followed by three successive baths of ethanol (100%, 5 min; 95%, 4 min; 70%, 3 min). After two baths of distilled water (2 × 2 min), the sections were incubated in antigen retrieval solution (Dako Target Retrieval Solution pH6) for 90 s at 95 °C in a deckloaking chamber (DC2012-220v, Biocare Medical). The sections were washed three times with PBS and incubated for one hour in antigen blocking solution (Dako) before overnight incubation at 4 °C with the following primary antibodies: rabbit anti-chromogranin A (CgA) (1/500; Atlas Antibodies, Sweden) or rabbit anti-5HT (1/200; Immunostar, USA) diluted in Dako Diluent (Dako Agilent). After three washes, sections were incubated for 2.5 h with Cy3-conjugated anti-rabbit IgG (1/500; Jackson ImmunoResearch, UK) at RT. The sections were then counterstained with DAPI (Sigma-Aldrich, USA), mounted in Prolong (Thermo Fisher Scientific, France), and viewed under a Zeiss AxioObserver fluorescent microscope (Zeiss). For each tissue section, the epithelium lining length was identified and calculated using Image J software. Next, along this lining, the number of 5-HT-immunoreactive (IR) or chromogranin A-IR cells was counted. Finally, the density of 5-HT or chromogranin A was calculated and expressed in number of cells per mm.

### Statistical analysis

Figures were plotted and statistical analyses were performed using the Prism software (GraphPad). Clinical and descriptive data were expressed as means (± standard deviations). Nonparametric tests were also conducted. Differences were considered statistically significant at *p* < 0.05. Correlation tests were performed using the one-tailed Spearman’s coefficient (nonparametric). In addition, outlier was identified using ROUT test. Only one outlier was identified and removed (Fig. 1C and G—outlier from the same patient).

### Ethics approval

This study was performed in line with the principles of the Declaration of Helsinki. Approval was granted by the Ethics Committee of Nantes University Hospital.

### Consent to participate

Informed consent was obtained from all individual participants included in the study.

### Supplementary Information

Supplementary Figure 1.

## Data Availability

The datasets generated during and/or analysed during the current study are available from the corresponding author on reasonable request.
