# Supplementary material for: Region-specific remodeling of the enteric nervous system and enteroendocrine cells in the colon of spinal cord injury patients
Source: Sci Rep. 2023 Oct 6;13:16902. doi: 10.1038/s41598-023-44057-y (PMC10558436; doi:10.1038/s41598-023-44057-y)
Supplement: Supplementary file 1 — Supplementary Figure 1. [file 41598_2023_44057_MOESM1_ESM.docx]

**Supplementary figure**

**
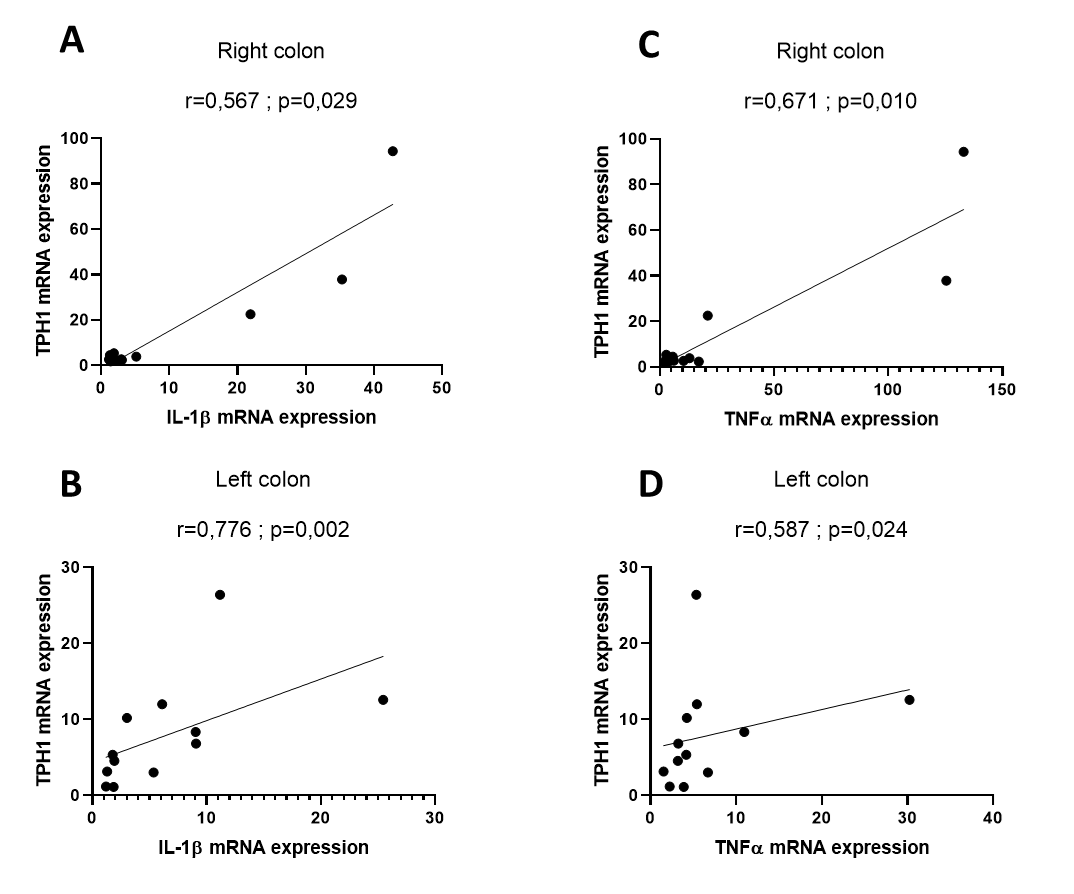
**

**Figure 1 (supplementary figure)**: Correlations between Tryptophan hydroxylase 1 (TPH1) mRNA expression and Interleukin-1 beta (IL-1β) mRNA expression in right (**A**) and left (**B**) colon of Spinal cord injury (SCI) patients, and between TPH1 mRNA expression and tumor necrosis factor alpha (TNFα) mRNA expression in the right (**C**) and left (**D**) colons of SCI patients. Spearman’s one-tailed, n=13 SCI patients.
